# Thoracic costotransverse joint pain patterns: a study in normal volunteers

**DOI:** 10.1186/1471-2474-9-140

**Published:** 2008-10-15

**Authors:** Brian A Young, Howard E Gill, Robert S Wainner, Timothy W Flynn

**Affiliations:** 1 Department of Physical Therapy, Sheppard Air Force Base, Texas, USA; 2 Physical Medicine and Rehabilitation, Wilford Hall USAF Medical Center, Lackland Air Force Base, Texas, USA; 3 Department of Physical Therapy, Texas State University, San Marcos, Texas, USA; 4 School of Physical Therapy, Regis University, Denver, Colorado, USA

## Abstract

**Background:**

Pain referral patterns of asymptomatic costotransverse joints have not been established. The objective of this study was to determine the pain referral patterns of asymptomatic costotransverse joints via provocative intra-articular injection.

**Methods:**

Eight asymptomatic male volunteers received a combined total of 21 intra-articular costotransverse joint injections. Fluoroscopic imaging was used to identify and isolate each costotransverse joint and guide placement of a 25 gauge, 2.5 inch spinal needle into the costotransverse joint. Following contrast medium injection, the quality, intensity, and distribution of the resultant pain produced were recorded.

**Results:**

Of the 21 costotransverse joint injections, 16 (76%) were classified as being intra-articular via arthrograms taken at the time of injection, and 14 of these injections produced a pain sensation distinctly different from that of needle placement. Average pain produced was 3.3/10 on a 0–10 verbal pain scale. Pain was described generally as a deep, dull ache, and pressure sensation. Pain patterns were located superficial to the injected joint, with only the right T2 injections showing referred pain 2 segments cranially and caudally. No chest wall, upper extremity or pseudovisceral pains were reported.

**Conclusion:**

This study provides preliminary data of the pain referral patterns of costotransverse joints. Further research is needed to compare these findings with those elicited from symptomatic subjects.

## Background

The thoracic spine has been described as the enigma within the vertebral column, with the diagnosis of pain originating from this region being historically problematic for the practitioner [1-5]. The neural complexity of the thoracic spine, along with referred visceral pain leads to poor pain source localization [6-8]. Research of thoracic spine pain referral patterns has been relatively sparse when compared to the cervical and lumbar spine regions[2,9-13], despite reports of equally disabling pain from this region[2,13-16].

The costotransverse and costovertebral joints are often suspected as sources of referred thoracic pain only after costly and often unnecessary negative visceral work-ups have been performed[2,17-20]. One example is T4 syndrome, a symptom complex originating in the upper thoracic spine and includes glove-like paresthesias of one or both upper limbs, referred pain into the neck and scapular regions, and a dull, aching generalized headache [21-23]. Successful treatment has been reported in case studies using manipulation and exercise intervention [21-23], despite the unknown cause of T4 syndrome. Both the thoracic intervertebral disks and thoracic zygapophyseal joints are thought to be primary pain generators in T4 syndrome based on their pain patterns, suggesting that dysfunction of the costotransverse joint may be implicated as well[13].

Costotransverse joints cannot be assumed to be a source of pain solely on the basis of pain mapping findings from other joints in the vertebral column[13]. Therefore, pain referral mapping in asymptomatic volunteers can provide information on the potential of the costotransverse joints to be a source of pain, and potentially to recreate clinically observed pain syndromes. This has been undertaken in thoracic zygapophyseal joints, where pain patterns have been documented in asymptomatic volunteers[13], as well as in subjects with thoracic pain[14]. Clinical pain patterns from the costotransverse joints have been hypothesized[3,20,24]. However, pain referral patterns for the costotransverse joints have yet to be definitively investigated. The suspected pain patterns from the costotransverse joints are likely similar to the thoracic zygapophyseal and costovertebral joints. Innervation of the costotransverse joints is from the lateral branch of the thoracic dorsal rami, whereas the thoracic zygapophyseal joints are innervated by the medial branches of the thoracic dorsal rami[8]. Costovertebral joints have been shown to receive sympathetic innervations from the neighboring sympathetic segment and the segment cranial to it[25]. Therefore, the purpose of this study was to identify and record the pain referral pattern of the asymptomatic costotransverse joint, and to stimulate further investigation of the costotransverse joints.

## Methods

### Subjects

Eight asymptomatic male subjects (36 years ± 7.3 years) without history of thoracic pain participated in the study. This study was approved by the Investigational Review Board at Wilford Hall Medical Center, Lackland Air Force Base, Texas. Informed consent was obtained from all subjects prior to participation. Pretest imaging studies of the costotransverse joints were not performed.

### Costotransverse Joint Selection

Subjects were allocated to receive consecutive, same-day right-sided T2, T4 and T6 costotransverse joint injections, or consecutive, same-day left-sided T3, T5 and T7 costotransverse joint injections. Subjects were not blinded to the level of injection, but were blinded to pain pattern responses in prior subjects.

### Fluoroscopy Guidelines

No duration of imaging was stated in the original description of technique reference[26]. Therefore, to minimize exposure to radiation, the total exposure to fluoroscopy was limited to 6 minutes or less per subject, as determined by the Wilford Hall Medical Center Radiation Safety Officer. This was calculated to provide the equivalent amount of radiation as 3.3 years of exposure to natural background radiation (7800 mR).

### Injection Procedures

The technique for injection has been previously described[26]. No sedation was utilized as was reported in the initial technique description[26]. Injections were performed with the patient in the prone position and not the prone oblique position as previously documented due to the inherent mobility of the C-arm fluoroscopy used in this study.

Once the subject was positioned prone, the skin overlying the target joint was prepped with betadine. Using intermittent video fluoroscopy, the target joint space was isolated and the point of needle insertion marked. Xylocaine^® ^(AstraZeneca LP, Wilmington, DE) (1.0%, 2 cc's) was then injected directly under the skin for topical anesthesia. A 25 gauge, 2.5 inch spinal needle was inserted into the underlying costotransverse joint guided by intermittent fluoroscopy toward the identified joint space. Imaging was performed in multiple angles (anteroposterior, as well as 30–45° oblique with a slight cephalic tilt) to guide needle advancement, and for verification of needle placement within the identified joint space as previously described by Dreyfuss[13]. The joint was then injected with ≤ 0.5 cc Omnipaque™ 240 (iohexol) Injection (contrast medium) (GE Healthcare Biosciences/Amersham Health, Piscataway, NJ) under constant imaging to distend the joint. Injection was continued until pain or pressurization of the capsule occurred allowing no additional contrast to be safely injected, or extracapsular spread of the contrast medium was noted by fluoroscopy[13]. An arthrogram was taken to document needle placement, joint selection, and for later data analysis.

### Outcome Measurements

#### Image Classification

All costotransverse joint arthrograms were analyzed to determine the extent of contrast within the joint, and thus to delineate between successful and unsuccessful joint injections. All images were analyzed by one investigator (HG). The following rating scale was utilized:

##### Good

An arthrogram which clearly outlines the extent of the joint and capsule.

##### Equivocal

An arthrogram which demonstrates some contrast within the joint, but does not clearly outline the extent of the joint or extravasates outside the joint.

##### Poor

Unable to determine if contrast is within the joint.

Successful joint injections were those rated as either good or equivocal.

#### Pain and Symptom Assessment

During each injection subjects were asked to distinguish between the sensations of the needle insertion/advancement and capsule distention. The numeric pain rating scale (0 = no pain; 10 = worst imaginable pain) was utilized to report the level of pain induced with capsular distension [27-29]. Subjects were also instructed to describe the pain/sensation induced and any referred pain, utilizing a list of pain descriptors, as well as self-selected descriptors.

#### Composite Pain Map Construction

The needle insertion point was circled and labeled with a skin marker, and the distribution of pain produced from the joint injection was also marked and labeled on the subject's skin by the injectionist via palpation and verbal interaction with the subject. Once the pain markings were complete, a digital photograph was taken of the pain distributions to allow accurate representation on a composite pain drawing. A separate investigator mapped the pain patterns on a body diagram. A composite pain map was then created from the individual joint maps.

## Results

**Figure 1:**
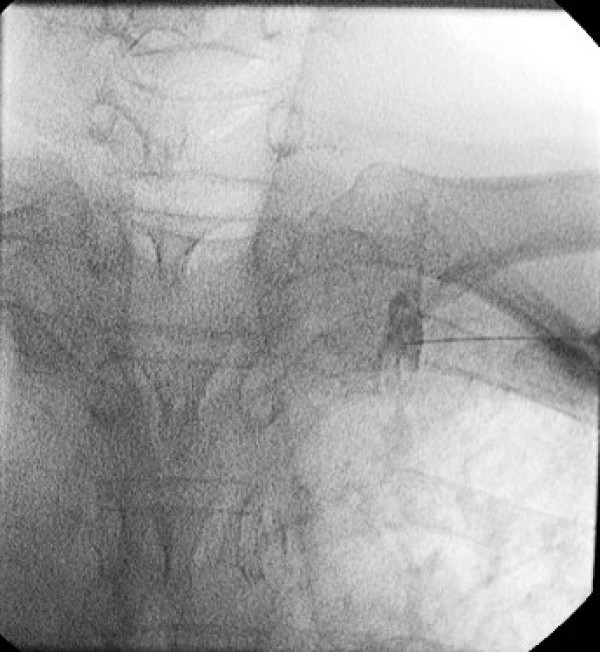
Image of good injection, right T2.

**Figure 2:**
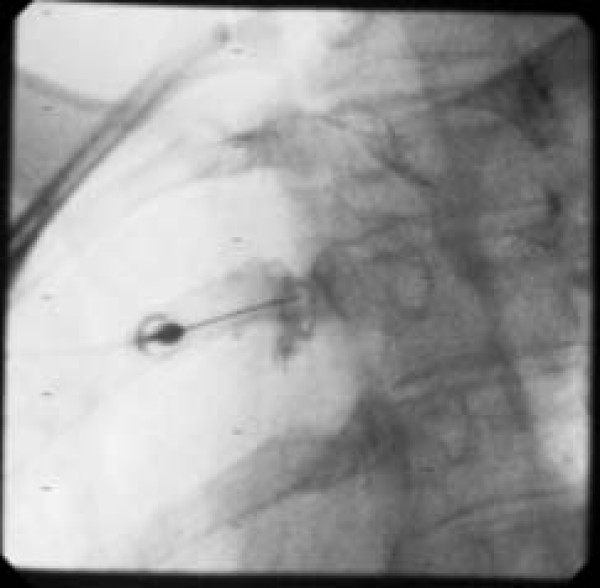
Image of equivocal injection, left T3.

**Figure 3:**
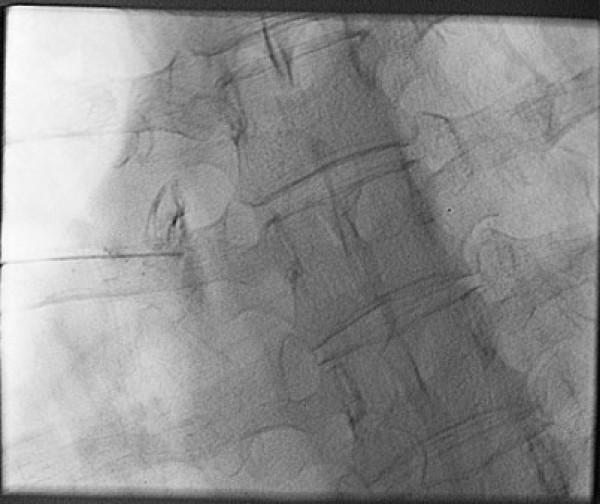
Image of poor injection, left T5.

No complications occurred in any subject from participation in this study. The mean radiation exposure time was 4.85 ± 1.03 minutes. Out of 24 potential costotransverse joint injections, a total of 21 injections were completed. The breakdown of the number of injections by joint and their classification, along with reasons for unperformed injections, are depicted in Table 1. Six arthrograms from the 21 completed costotransverse joint injections (29%) were classified as good, and 10 (47%) were classified as equivocal. Extracapsular spread of the injected medium was one reason to terminate further injection into the joint. As there were no differences in the pain pattern reported for those joints rated as good and equivocal, and there was evidence of intracapsular injection prior to the extracapsular spread, the good and equivocal groups were therefore combined into a "successful" injections category for the remainder of the analysis. The remaining 5 (24%) joint arthrograms were classified as poor, giving our accuracy of needle placement into the costotransverse joint utilizing fluoroscopy as 76%. Examples of each classification are presented in figures 1, 2, 3.

**Table 1:** Frequency of costotransverse joint injections and ratings.

	Right T2	Right T4	Right T6	Left T3	Left T5	Left T7
# injections attempted	3	4	2	4	4	4
# injections not performed	1^†^		2^‡^			
# Good injections	2	0	0	1	1	2
# Equivocal injections	1	3	2	3	1	0
# Poor injections		1			2	2

^† ^Difficulty visualizing joint space due to scoliosis.

^‡ ^One due to imaging time constraints; one due to inability to visualize costotransverse joint on fluoroscopy.

Of the 16 successful costotransverse joint injections, 14 (88%) of these injections produced a sensation during capsular distension distinctly different from that of needle placement. One left T3 injection, and one right T4 injection did not produce a pain sensation distinctly different from needle placement. The average pain from capsular distension for the 14 symptom producing injections was 3.3 ( ± 1.8) on the 0–10 numeric pain rating scale.

**Figure 4:**
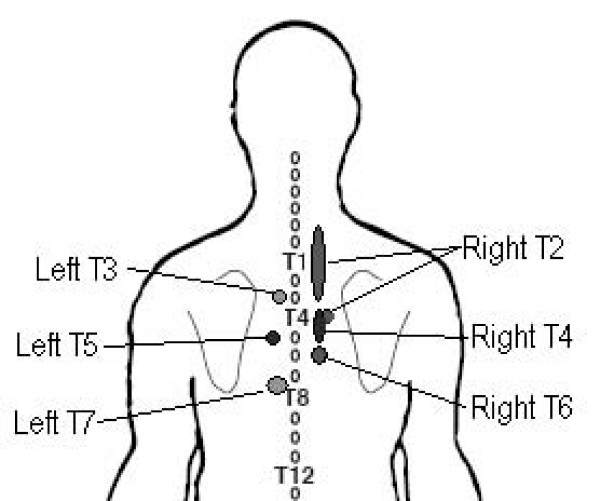
Composite diagram of costotransverse joint pain patterns.

The individual pain patterns from those 14 costotransverse joint injections which produced a distinct capsular distension sensation were combined to create the costotransverse joint composite pain map (Figure 4). In general reports of pain sensations were ipsilateral, and remained local to the target joint. Only pain elicited from the right T2 injections appeared to refer approximately 2 vertebral segments superior and inferior from the target joint. One subject did note tightness across the abdomen at the level of the xyphoid process with a right T6 injection. Provoked symptoms were described generally as a deep, dull ache and pressure sensation, with one subject describing a left T5 joint provoked pain as a sharp, burning pressure, and another left T5 described as a sharp pressure. The average volume of contrast medium injected was 0.4 cc (SD ± 0.1 cc).

## Discussion

This is the first study to attempt to document the pain referral patterns of asymptomatic costotransverse joints. We have initiated this process as outlined by Dwyer and colleagues[11]: first, a joint should produce pain when stimulated in normal volunteers; second, in patients with similar forms of pain, the pain should be relieved with anesthetization.

Due to the invasive nature and risk of this type of investigation, we limited this study to a small number of asymptomatic subjects to determine preliminary pain patterns[11,13]. From the small number of joints stimulated, it appears that there is a reproducible pattern and sensation of pain from asymptomatic costotransverse joints. This pattern was local to the target joint, and consistent with Hilton's Law, which states that the innervation of a joint is the same innervation as the muscles which move the joint and the skin overlying the joint.

The provoked pain patterns significantly overlap the pain patterns described in prior studies stimulating other spinal and soft tissue structures[9,13,14]. Therefore, pain patterns are unreliable in diagnosis. Further investigative work to identify symptomatic costotransverse joints will need to be performed to both stimulate and then anesthetize these joints in patients presenting with thoracic pain[10,30]. This procedure will not only aid in validation of our findings in symptomatic patients, but will also lay the foundation for therapeutic costotransverse joint injections. With a 34–48% prevalence of thoracic zygapophyseal joint pain, and a 42–58% false-positive rate [31-33], it is anticipated that a large number of patients would be required to ascertain true costotransverse joint data.

Two of the successful costotransverse injections (12%) did not provoke a sensation upon capsular distension that was distinguishable from needle insertion/placement. Dreyfuss[13] reported non-painful response to capsular distension in 27% of thoracic zygapophyseal joint injections in asymptomatic subjects. This non-painful response may have been due to an insufficient amount of contrast medium being injected to cause capsular distention[11,13], or due to the use of a non-irritating injection agent. Although Lau[26] reported injecting a total of 1.7 cc of fluid into the costotransverse joint when describing the costotransverse joint injection technique, no studies have reported on the available volume for this joint. Since the costotransverse joints are anatomically smaller than the zygapophyseal joints, we utilized the amount of fluid injected into the zygapophyseal joints[13] as a baseline for estimating the volume limit for the costotransverse joints. Dreyfess[13] injected between 0.4 to 0.6 ml. Therefore, we elected to limit the volume injected into the costotransverse joint to no greater than 0.5 cc as a precaution to prevent rupturing the joint capsule from overpressurization. Perhaps this amount of contrast was insufficient to cause adequate capsular distension in two of our costotransverse joints to provoke a symptomatic response. However, only 12% of our joints were asymptomatic as compared to 27% of zygapophyseal joints, perhaps suggesting our volume selection was appropriate due to the smaller size of the costotransverse joint compared to the zygapophyseal joint.

We used a non-irritating contrast agent in an attempt to provoke symptoms from capsular distension, similar to the state of joint effusion rather than chemical irritation. However, inflammatory cyctokines released from joint tissue irritated from needle insertion[34], stimulation of joint capsule nerve endings during needle penetration, and irritation of the joint synovium may have been other sources of elicited symptoms. Our interest was the symptom produced upon the injection of contrast medium into the joint, and patients were asked to distinguish this sensation from that of needle placement. Approximately 1–2 minutes lapsed between the needle placement and the injection of contrast medium, as the needle placement was verified by fluoroscopy from two imaging angles. Non-contrast agents have been used extensively in prior pain pattern studies[11,13,35], and have been successfully used to stimulate symptomatic thoracic zygapophyseal joints[14] in an effort to reproduce thoracic pain. Had hypertonic saline been utilized, a potentially more noxious stimulus, the pain referral patterns observed may have been broader in range, more intense, or other clinically reported symptoms may have been provoked[11,35].

Five of our arthrograms were rated as poor, and thus not included in our analysis. Although we attempted to verify needle placement in each joint before the injection of contrast medium, the intricate anatomy of the costotransverse joint may have been the biggest limitation of our study, possibly limiting the ability to fully place the needle within the joint space. The costotransverse joint is the synovial articulation between the rib tubercle of typical ribs and the vertebral transverse process[36]. The narrow costotransverse joint space is surrounded by a thin articular capsule and strong costotransverse ligaments which tightly bind the joint and limit mobility to slight gliding motions. It is bounded laterally by the rib tubercle and posteriorly by the transverse process, which greatly limits its accessibility. This study is additionally limited by the intricate biomechanical relationship between the costotransverse and costovertebral joints[37], adding further complexity to the diagnosis of thoracic pain. Finally, it is well documented that pain referral patterns of the spine are insufficient in determining the exact source of pain, because of their overlap[7]. More specific diagnosis and treatment approaches are needed, such as the use of medial branch blocks in the evaluation of potential thoracic zygapophyseal joint mediated pain[30].

Further studies exploring the pain patterns of the costotransverse joint are needed to validate these findings in symptomatic patients. One possibility would be to stimulate and then anesthetize the costotransverse joints in patients presenting with this pain pattern to determine response, as has been performed in thoracic zygapophyseal joints[14]. Validation of provoked pain patterns has been performed in the cervical spine, demonstrating that the evoked patterns in normal volunteers can be clinically accurate[10]. In developing further studies, alternative imaging techniques for these injections should be considered in an attempt to minimize exposure to radiation. Ultrasound-guided facet injections have initially been studied for cervical[38] and lumbar[39] facets.

## Conclusion

This study provides preliminary data on the pain referral patterns of the costotransverse joints. From the small number of joints stimulated, it appears that there is a reproducible pattern and sensation of pain from asymptomatic costotransverse joints.

## Competing interests

The authors declare that they have no competing interests.

## Authors' contributions

All authors contributed to project conception and design. HG and BY performed data acquisition. All authors contributed significantly to data analysis/interpretation, and drafting/revising the manuscript. All authors have read and approved the final manuscript.

## Pre-publication history

The pre-publication history for this paper can be accessed here:


